# Potentiated early neural responses to fearful faces are not driven by specific face parts

**DOI:** 10.1038/s41598-023-31752-z

**Published:** 2023-03-21

**Authors:** Maximilian Bruchmann, Léa Mertens, Sebastian Schindler, Thomas Straube

**Affiliations:** 1 Institute of Medical Psychology and Systems Neuroscience, University of Muenster, Von-Esmarch-Str. 52, 48149 Münster, Germany; 2 Otto Creutzfeldt Center for Cognitive and Behavioral Neuroscience, University of Muenster, Münster, Germany

**Keywords:** Neuroscience, Physiology, Psychology

## Abstract

Prioritized processing of fearful compared to neutral faces is reflected in increased amplitudes of components of the event-related potential (ERP). It is unknown whether specific face parts drive these modulations. Here, we investigated the contributions of face parts on ERPs to task-irrelevant fearful and neutral faces using an ERP-dependent facial decoding technique and a large sample of participants (N = 83). Classical ERP analyses showed typical and robust increases of N170 and EPN amplitudes by fearful relative to neutral faces. Facial decoding further showed that the absolute amplitude of these components, as well as the P1, was driven by the low-frequency contrast of specific face parts. However, the difference between fearful and neutral faces was not driven by any specific face part, as supported by Bayesian statistics. Furthermore, there were no correlations between trait anxiety and main effects or interactions. These results suggest that increased N170 and EPN amplitudes to task-irrelevant fearful compared to neutral faces are not driven by specific facial regions but represent a holistic face processing effect.

## Introduction

It is of paramount importance to detect threat signals and prioritize the processing of such emotional over neutral information^1,2^. In line with this notion, electrophysiological (for a review, see^3^) or brain imaging studies (for a review, see^4^) showed enhanced brain responses to threat-associated vs. neutral stimuli. For example, prioritized processing of fearful compared to neutral faces is reflected in increased amplitudes of different components of the event-related potential (ERP), such as the P1, N170, and EPN (for reviews, see^5–7^). One important question is, what information exactly leads to the potentiation of specific ERP components in response to specific faces or facial expressions. Low-level visual features such as spatial frequencies^8–11^ or differences in specific face parts such as the eyes^12–15^ have been proposed to drive specific ERP components. On the other hand, several studies show that threat-associated faces potentiate early ERPs despite the control or absence of visual differences between negative and neutral faces^16–18^.

A second question is whether interindividual differences modulate the effects of facial expressions. One important trait related to altered threat detection and perception is trait anxiety, which has been proposed to lead to hypervigilant processing of threat-related stimuli^19–22^. However, while early studies reported increased early ERPs for fearful faces in high trait-anxious participants^23–25^, other studies found no effect^26,27^ or even attenuated differential ERPs^28,29^. Thus, although theoretical arguments (e.g.,^21,23^) strongly suggest a relation between trait anxiety and early neural responses to threat, findings are inconclusive. Further research is strongly needed to investigate the role of trait anxiety and facial features for the increased processing of fearful faces during different processing stages indexed by specific components of the ERP.

The earliest of the relevant components in this regard is the occipitally scored P1, which is thought to reflect early stages of stimulus detection and discrimination^30–32^, being enlarged for faces compared to objects^33,34^. Findings on P1 modulations by fearful faces are mixed (for a review, see^7^), with studies reporting larger amplitudes for fearful compared to neutral faces^35–38^ while others do not show such differences (e.g.,^24–27,39^). Recent studies show that ERP modulations during the P1 interval strongly depend on low-level visual information^8,40,41^.

The subsequent N170 is viewed as a structural encoding component and reliably found to be enlarged for faces compared to objects^42^. Fearful expressions have been shown to increase further this ERP component^6^. Notably, increased N170 amplitudes by emotional expressions are not explained by low-level information^8,40,43^ and are highly resistant to various attentional manipulations^27,37,40,44–47^. Both holistic accounts^47–50^ and accounts assuming a role of specific features, such as mouth^51–54^ or eyes^53–56^ have been proposed to explain N170 modulations by faces in general and fearful facial expressions in particular.

The EPN peaks between 200 and 300 ms and is observed as a differential negativity when contrasting emotional and neutral stimuli, including faces. It has been repeatedly found to be increased for fearful compared to neutral expressions^29,57–59^ and has been linked to early attentional selection^60^. A previous study showed that the EPN increase for fearful faces is affected by specific spatial frequencies^8^.

Several aspects of face processing have been shown to be sensitive to manipulations of the spatial frequency spectrum, typically by presenting frequency-filtered faces^9–11,61–63^. The P1 has been shown to be enhanced for low spatial frequency (LSF) faces compared to high spatial frequency (HSF) faces^10,11^. Results are less clear regarding the N170, where some studies reported smaller N170 amplitudes for LSF compared to HSF faces^10,11^, whereas another study did not find amplitude differences, but an increase in N170 peak latency for HSF compared to LSF faces. Furthermore, the inversion effect on the N170 (i.e., larger amplitudes for inverted compared to upright faces) was no longer observed when low spatial frequencies were removed from the faces^9^. We conclude that spatial frequencies affect early evoked potentials to faces, although their effect regarding the N170 is disputed.

Regarding the interplay of spatial frequency and emotional expressions, it is essential to note that fearful compared to neutral faces contain more spectral power in low spatial frequencies, most prominently in the eye region^64^. We recently showed that controlling for this natural confound does not alter N170 differences between fearful and neutral faces but has subtle effects on the P1 and the EPN^8^.

Using facial decoding methods, which allow mapping the effect of specific face parts on ERP modulations^65^, some studies suggest that specific facial regions contribute to N170 ERP modulations^53,54,65–68^. The eye region, in particular, played an important role for fearful facial expressions. More contrast in this region led to better emotion recognition performance^54,69^ and to enhanced N170 amplitudes^66,68^. While these studies yielded precise individual estimates of facial decoding maps with small samples (N = 3^51^; N = 3^63^; N = 2^64^; N = 2^65^), no statistical inferences about a population or relationships to interindividual differences, such as trait anxiety, were possible. Furthermore, these studies required participants to identify the emotional expression on each trial, creating specific attentional demands for participants. Task relevance has been shown to affect ERPs, especially the EPN and later components, to facial expressions and the differences between them^15,27,37,70^. Although task relevance is a valid setting in which to study effects of facial expressions on ERPs, we suggest that task irrelevant faces yield a more default-like and thus ecologically more valid insight into emotional face processing. The studies mentioned above leave room for speculation whether differences in specific ERP components between fearful and neutral faces are mainly driven by specific face parts or represent a response to the whole face if it is task irrelevant.

Taken together, it remains to be investigated to what extent the potentiation of early and mid-latency components of the ERP is driven by specific face parts or an interaction of face parts and spatial frequency. Furthermore, whether effects are related to interindividual differences, such as trait anxiety, is unknown.

The present pre-registered study (osf.io/n72w3) investigated contributions of face parts on ERPs to task-irrelevant fearful and neutral faces, using a facial decoding technique and a large sample of participants (N = 83) varying in trait anxiety scores. We assumed that at the level of the P1, modulations might be mainly driven by image intensity, irrespective of the facial region. This modulation should be most pronounced in mid-range spatial frequencies (between 3.75 and 7.5 cycles per degree of visual angle; cpd), reflecting the properties of the human contrast sensitivity function (CSF^63^). In contrast, we hypothesized the eye region to modulate N170 amplitudes. Finally, for the EPN, we predicted that diagnostically critical face-feature (eyes, mouth) should drive fearful vs. neutral differences. Based on our observation that emotion effects on the EPN interact with spatial frequency^8^, we assumed these effects also depend on spatial frequency. In addition to these analyses, we exploratively investigated whether effects are modulated by interindividual differences in trait anxiety.

## Methods

### Participants

According to the registered data sampling plan, 87 participants were examined (19 male, 68 female). Similar decoding approaches to this study have been used in the context of group statistical analyses^71,72^, however, not related to emotional expressions. Therefore, power calculations were not possible. We chose the highest sample size suitable for our human and financial resources to maximize sensitivity. Four participants were excluded due to bad EEG data, resulting in 83 participants (19 male, 64 female). Participants gave written informed consent and received 10 Euros per hour for participation. Inclusion criteria required participants to have normal or corrected-to-normal vision and no reported history of neurological or psychiatric disorders. On average, participants were 23.62 (SD = 3.68; min = 19; max = 36; median = 23; see Fig. 2) years old. The study was approved by and conducted following the guidelines of the Ethics Committee of the University of Münster (Germany; vote ID 2018–705-f-S).

### Apparatus and stimuli

The facial stimuli were taken from the Radboud Faces Database^73^. For these stimuli, the position of the eyes and head orientation are well standardized. We converted the faces into greyscale and cut out the oval center of each face, removing facial hair. Thirty-six identities (18 male, 18 female) were used, displaying either fearful or neutral expressions in frontal viewing angle. Stimuli were created by adapting the so-called bubble technique^65^.

**Figure 1:**
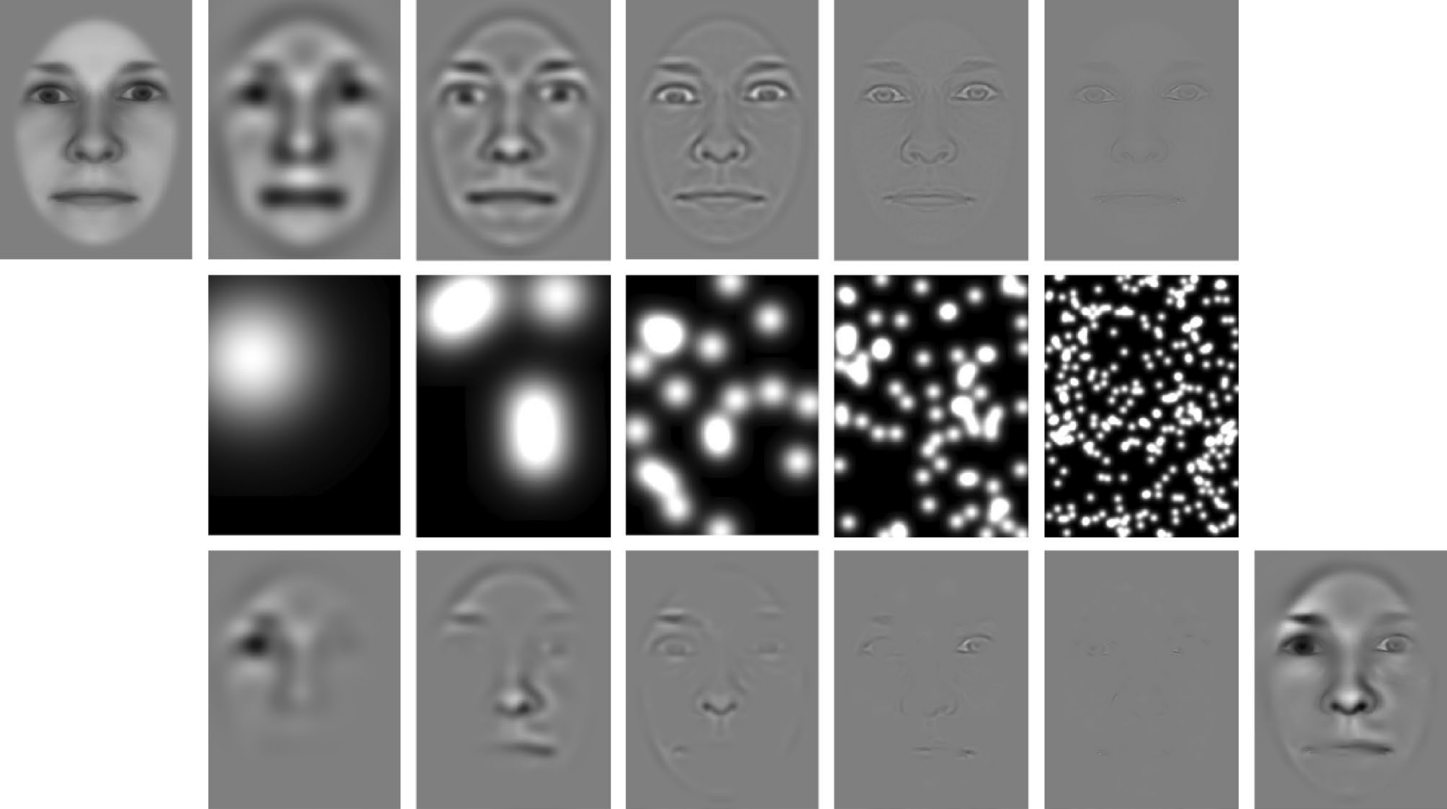
Illustration of our adaptation of the bubble technique^65^. The top row shows the spatial frequency decomposition of a single (i.e., the leftmost) image. The middle row depicts randomly placed Gaussian blobs for each spatial scale. The total surface covered by all blobs is kept constant. The bottom row shows the decomposed faces, masked by the corresponding blobs. The final (i.e., the rightmost) image shows a stimulus example, which contains the sum of all images depicted in the bottom row. Please note that all face images shown in this publicatin do not belong to the actual stimulus set. For purposes of copyright protection we used an artificially created face (generated using the software FaceGen 3D Print Home 2.0) here and as a background image for all classification maps (Figs. 4, 6, 8, 9).

In contrast to classical ERP studies of facial expressions, this technique aims at relating single-trial amplitudes to pixel-wise variations in image contrast across a comparably higher number of stimuli (500–2000) instead of averaging ERPs across several repetitions (e.g., 50–100) of stimuli from the same category. The stimuli consist of fractions of facial information obtained by randomly placing Gaussian blobs (“bubbles”) on facial images, with the bubbles determining the contrast at a given image location. More specifically, the face images are first decomposed into separate spatial frequency scales (see Fig. 1, top row). Bubbles are then placed per scale with a constant blob-size-to-scale ratio (see Fig. 1, middle row). The final image on a given trial consists of the same amount of information per scale but is randomly scattered across the image (see Fig. 1, bottom right image).

Instead of ERPs per stimulus category, this technique yields classification images, i.e., “face maps”, showing which face regions contributed to amplitude modulations of a given ERP component (details see below). Classification images can be obtained across emotional categories, showing face regions that amplify or diminish the ERP component separately for each spatial scale. They can also be obtained per emotional category and then compared, showing face regions that contribute to amplitude differences separately for each spatial scale.

Spatial scales were separated by convolving each image with Gabor gratings with 12 different orientations ranging from 0° to 165° in steps of 15°. The frequency bands were identical to those by Gosselin and Schyns^65^, i.e., 3.75 to 7.5, 7.5 to 15, 30 to 60, and 60 to 120 cycles per image (cpi). However, we excluded the lowest frequency band (1.875 to 3.75 cpi) as it contained no detectable information, most likely due to a different face image choice than Gosselin and Schyns^65^. With faces presented at a bizygomatic diameter of 6.2 deg of visual angle, the average spatial frequencies of each band are 0.67, 1.3, 2.7, 5.4, and 10.7 cpd.

Each frequency band was sampled with four equidistant frequencies. The resulting images were averaged across the 12 orientations and four frequencies per band (see Fig. 1 top row). Bubbles were created by randomly positioning Gaussian blobs with standard deviations corresponding to the width of 0.5 cycles of the respective average bandwidth (e.g., with 90 cycles per image for the average of the finest scale, the standard deviation of the Gaussian blob corresponded to the image height divided by 180). Thus, from -3 to + 3 standard deviations of the Gaussian window, 3 complete cycles were included per bubble at each scale, corresponding to Gosselin and Schyns^65^. The total energy of all Gaussian blobs on each scale was equal to 80% of a complete Gaussian blob at the coarsest scale (see Fig. 1 middle row). After applying the Gaussian windows to each scale, the resulting image intensities were summed. We calculated the minimal and maximal luminance across all images and scaled all resulting images with a single constant factor so that all final images contained the same average gray background while maximizing the range of gray levels from black to white without clipping. To ensure attention to the screen, target stimuli were generated by applying the same filtering and bubble procedure to white noise images, to which the same oval cut-out was applied. In total, 600 different images per emotional expression and 100 different target stimuli were created. This procedure was repeated four times to obtain four differently randomized experiment versions, counterbalanced across subjects.

### Procedure

The experiment was programmed and ran with Matlab (Version R2019b; Mathworks Inc., Natick, MA; http://www.mathworks.com), the Psychophysics Toolbox (Version 3.0.16)^74,75^, and the Eyelink Toolbox^76^. In each trial, a fixation mark was presented of a randomized duration between 300 and 700 ms, followed by a bubble stimulus for 50 ms, followed by a blank screen presented for 500 ms before the next fixation mark was presented. All stimuli were presented at the center of the screen. Participants were instructed to avoid eye movements and blinks during the stimulus presentation. To ensure that participants paid attention to the presented faces, Eye-tracking was used (EyeLink 1000, SR Research Ltd., Mississauga, Canada), pausing the presentation of faces when their gaze was not directed to the center of the screen, defined by a circular region with a radius of 0.7° around the fixation mark. If a gaze deviation was detected for more than five seconds despite a participant's attempt to fixate the center, the eye- tracker calibration procedure was automatically initiated.

Additionally, participants were instructed to respond to a target trial by pressing the space bar. Emotion conditions and target trials appeared in randomized order. Including breaks, the experiment lasted approximately 45 min. After testing, participants were asked about the effort and difficulty of the experiment, and tiredness during and after the experiment. Further, they responded to a demographic questionnaire, the BDI-II^77^ and STAI Trait questionnaire^78^, and a short version of the NEO-FFI^79^. For the current study, only the STAI was analyzed.

### EEG recording and preprocessing

EEG signals were recorded from 64 BioSemi active electrodes using Biosemis Actiview software (www.biosemi.com). Four additional electrodes measured horizontal and vertical eye movement. The recording sampling rate was 512 Hz. Offline data were re-referenced to average reference, and band-pass filtered from 0.01 Hz (6 dB/oct; forward) and low-pass with a cut-off frequency of 40 Hz (24 dB/oct; zero-phase). Recorded eye-movement were corrected using the automatic eye-artifact correction method implemented in BESA^80^. Filtered data were segmented from 100 ms before stimulus onset until 1000 ms after stimulus presentation. Baseline correction used 100 ms before stimulus onset.

### EEG data analyses

Components of interest were scored individually by inspecting each subject’s evoked potential averaged across all 1200 face stimuli. The search criteria for the components of interest were characterized as follows: P1: a bilateral positive peak at an occipital/occipito-parietal region of sensors (search space: Iz, Oz, O1, O2, PO3, PO4, PO7, PO8, P3, P4, P5, P6, P7, P8, P9, P10) in the time range between 80 to 130 ms after stimulus onset. The interval of interest was defined as ± 10 ms around the peak. N170: a bilateral negative peak at a more temporal region of sensors (search space: O1, O2, PO7, PO8, P7, P8, P9, P10, TP7, TP8, TP9, TP10) in the time range between 120 to 180 ms after stimulus onset. The interval of interest was defined as ± 10 ms around the peak. EPN: a bilateral negative peak following the P2 peak at temporal sensors (same search space as N170) in the time range between 200 to 350 ms after stimulus onset. The interval of interest was defined as ± 50 ms around the peak.

After defining the individual sensors and intervals of interest, the average amplitude at these sensors and intervals was calculated per trial using the per-trial activation of -100 to 0 ms relative to stimulus onset as the baseline. Following Smith and colleagues^69^ procedure, we derived classification images for each subject as follows: Images were re-scaled from 800 × 592 px to 200 × 148 px to reduce computation time. For each participant and each component of interest, trials were sorted by amplitude. We then labeled trials above the individual 60th percentile and below the 40th percentile as high and low-amplitude trials, respectively. We calculated the average image intensity from the maps of randomly positioned Gaussian blobs (see Fig. 1 middle row) per trial, separately for each pixel and spatial scale, separately for high and low-amplitude trials. The procedure was performed once across all stimuli irrespective of their emotional category to obtain total classification images comparable to previous studies^53^. Additionally, classification images were calculated per emotional category by applying the above-described procedure separately to trials with neutral and fearful faces. This calculation resulted in one low- and one high-amplitude map per subject, component of interest, spatial scale, and emotional category (total, neutral, fearful).

Please note that the classification image approach deviates from the pre-registered protocol, which was based on correlations between single-trial amplitudes and image intensities. We opted for replicating the methodology of previous studies for the sake of direct comparability.

Classification images, i.e., the differences between low- and high-amplitude maps, were statistically analyzed using cluster-based permutation (CBP) analyses. CBPs based on cluster mass were performed by running paired t-tests per pixel of each map. Pixels were labeled as significant if *p* < .01 (i.e., pixel-α = .01). Clusters were defined as significant orthogonally neighboring pixels, and the cluster mass was calculated by summing all t-values within a cluster, separately for positive and negative t-values. Cluster masses were then compared to the distribution of maximum cluster masses obtained from 5000 permutations based on sign-flipping, i.e., the multiplication of each high/low difference with randomly -1 or 1. Clusters with a mass exceeding the 99th percentile of the permutation distribution were defined as significant (i.e., cluster-α = .01). The procedure was performed for classification maps based on all trials, for classification maps based on neutral and fearful faces separately, and once for the difference between classification maps of fearful and neutral faces.

In case of absent significant differences between classification maps of fearful and neutral faces, we performed Bayesian analyses to quantify the likelihood of the absence of differences. For this purpose, we used the clusters observed in the classification images across all trials as effect masks for fearful and neutral classification images, averaging their intensity within this region per emotional category. For these analyses, the null hypothesis was specified as a point-null prior (i.e., standardized effect size δ = 0) and defined the alternative hypothesis as a Jeffreys-Zellner-Siow (JZS) prior, i.e., a folded Cauchy distribution centered around δ = 0 with the scaling factor r = 0.707. This scaling factor assumes a roughly normal distribution. To assign verbal labels to the strength of evidence, we followed the taxonomy suggested by Jeffreys^81^, labeling Bayes factors with a BF_01_ of 1 as no evidence, BF_01_ between 1 and 3 as anecdotal evidence, 3–10 as moderate evidence, 10–30 as strong evidence, 30–100 as very strong evidence, and larger BFs as extreme evidence in favor of the null hypothesis.

Possible correlations between individual traits anxiety scores and classification maps were explored by performing a CBP as described above, except that instead of calculating t-tests per pixel, we calculated Pearson correlations between the high-vs-low-amplitude maps and individual STAI scores. Random permutations were generated by randomizing the assignment of difference maps to the individual STAI values. Correlation analyses were restricted to the difference in classification maps between fearful and neutral faces. Please note that three participants chose not to complete the questionnaires. Therefore, the analyses of STAI correlations are based on the data from 80 participants.

Additionally, to the single-trial-based analysis with individualized component identification, we performed a standard ERP analysis to compare the average amplitudes per component of interest between all images in the fearful and neutral categories. For the purpose of illustration we calculated the 95% bootstrap confidence interval^82^ for the differential ERPs (fearful–neutral) based on 1000 samples.

## Results

### Behavior

**Figure 2:**
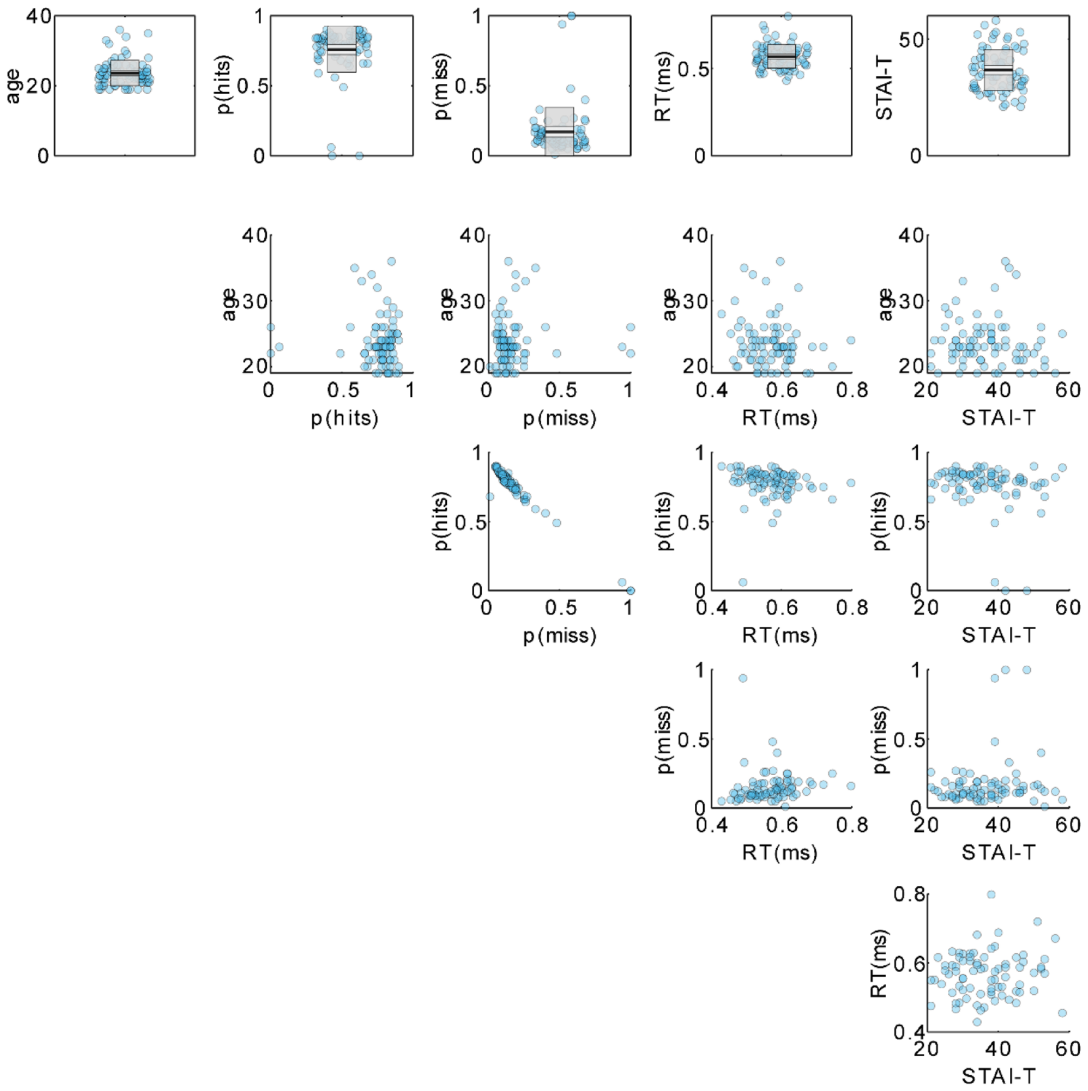
Demographic and behavioral data. Top row: univariate scatterplot of age, relative frequencies of hits and misses, average reaction times, and trait anxiety scores. Random jitter in x-direction was added to reduce cluttering. The superimposed gray boxes illustrate ± 1 standard deviation (SD; dark gray) and ± 1 standard error of measurement (SEM; light gray) around the mean (black line). Rows 2 to 5 contain scatterplots of each pairwise combination of these variables.

On average, subjects detected 76.51% (SD = 15.48%) of the targets and made 1.5% false alarms (SD = 1%). The average reaction time was 561 ms (SD = 65 ms). Trait anxiety scores (STAI-T) ranged between 21 and 58 (mean = 36.763, SD = 8.809). Individual behavioral data, demographic data, and trait anxiety scores are illustrated in Fig. 2.

### ERPs

#### P1

**Figure 3:**
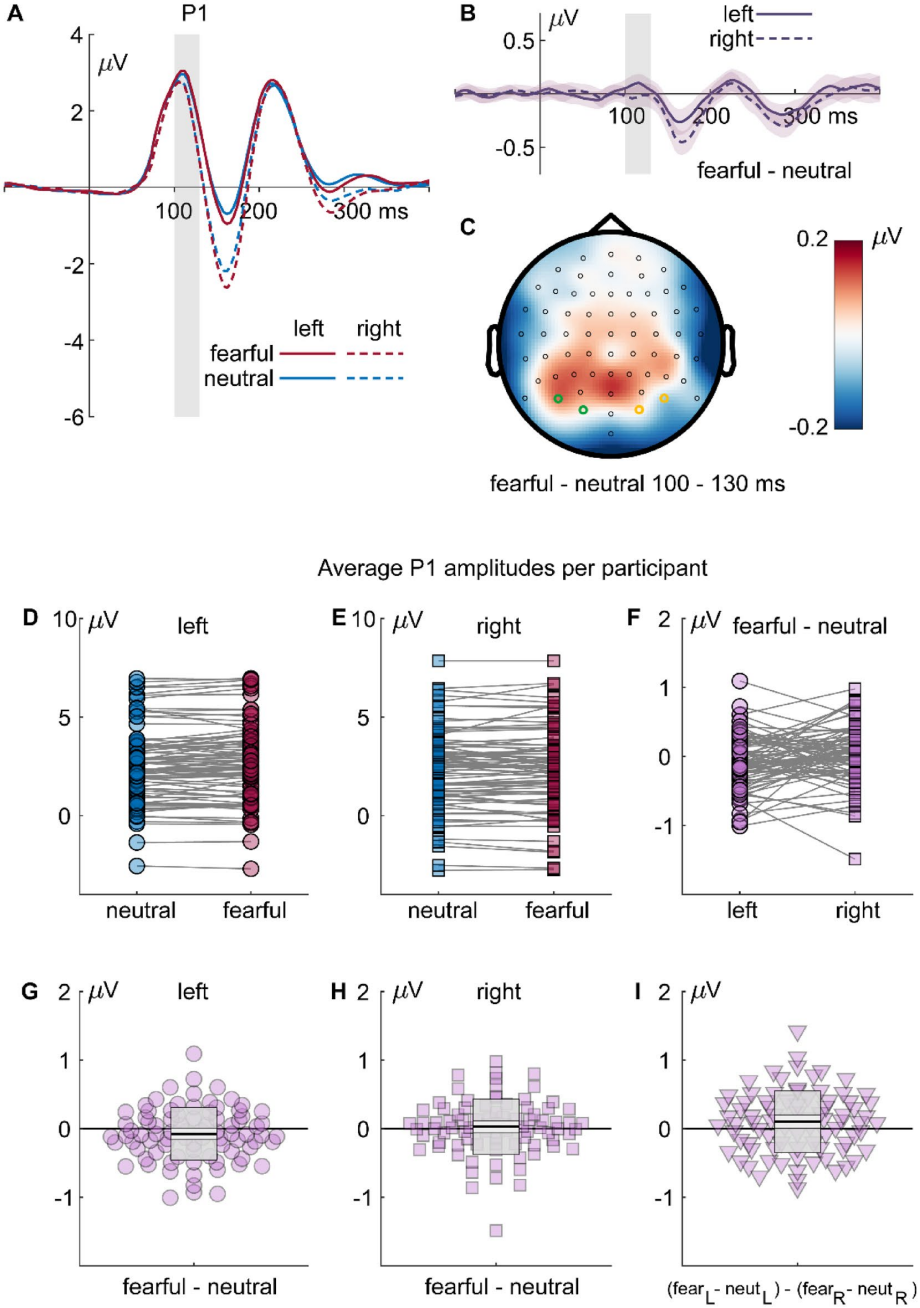
Results for the P1 component: (**A**) ERP waveforms for all fearful (red) and neutral (blue) expressions, separate for left (solid lines; PO7 and O1) and right (dashed lines; PO8 and O2) electrodes. (**B**) Differential ERPs (fearful–neutral; purple), separate for left (solid lines; PO7 and O1) and right (dashed lines; PO8 and O2) electrodes. The gray rectangles mark the interval of interest used for calculating average amplitudes and for the topography. Shaded areas around the differential ERPs depict the 95% bootstrap confidence intervals around the mean difference. (**C**) Topography of the differences in the highlighted interval of interest. (**D**–**F**) individual average P1 amplitudes or amplitude differences, respectively. (**G**, **H**) Individual differences between fearful and neutral faces and (**I**) individual left/right differences of fearful/neutral differences. The dots are horizontally displaced proportional to the local density to reduce cluttering. The superimposed gray boxes illustrate ± 1 standard deviation (SD; dark gray) and ± 1 standard error of measurement (SEM; light gray) around the mean (black line).

The ANOVA of mean ERPs across all spatial scales and bubble stimuli, revealed a significant interaction of emotion and hemisphere, F_(1,82)_ = 4.247, *p* = .042, partial *η*^2^ = .049. As Fig. 3a–c indicates, amplitude differences between fearful and neutral faces were more pronounced in the left than in the right hemisphere. Figure 3d–i additionally illustrates individual amplitudes and amplitude differences.

**Figure 4:**
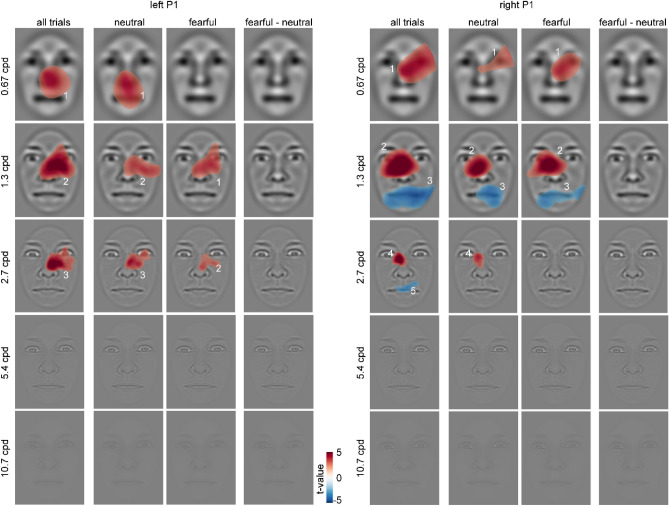
Classification images for the P1 component depicting face regions that contributed significantly to an amplitude increase (red) or decrease (blue). The clusters are enumerated to assign them to the respective statistics reported in the main text. Please note that the electrodes used for classification image calculations were chosen individually (see “Methods” section), whereas the ERP data in Fig. 3 are based on common sensors for illustration.

Classification maps for the left-hemispheric P1 of all trials combined show positive clusters (increased P1 amplitudes) in the region of the nose and the inner canthus of the right eye, restricted to spatial frequencies between 0.67 and 2.7 cpd (cluster masses (C) from lowest to highest frequency: C_1_ = 12,334.8, *p* < .001; C_2_ = 15,374.2, *p* < .001; C_3_ = 8473.4, *p* < .001; see Fig. 4). For neutral faces, significant positive clusters were observed in the region of the nose, the mouth and the inner canthus of the right eye, restricted to spatial frequencies between 0.67 cpd and 2.7 cpd (C_1_ = 13,494.3, *p* < .001; C_2_ = 8737.5, *p* < .001; C_3_ = 5003.9, *p* < .001). For fearful faces, the maps show positive clusters in the region of the nose and the right eye for spatial frequencies between 1.3 and 2.7 cpd (C_1_ = 9532.5, *p* < .001; C_2_ = 3012.4, *p* < .001). There were no significant difference between classification maps of fearful and neutral faces. We calculated Bayesian t-tests as described in the Method section. For all three clusters found across all trials for the left P1, we observed moderate evidence for the null hypothesis (C_1_: BF_01_ = 6.932, C_2_: BF_01_ = 7.459, and C_3_: BF_01_ = 7.611).

For the right hemispheric P1, classification maps of all trials show positive clusters around the right eye at a spatial frequency of 0.67 cpd. At 1.3 cpd and 2.7 cpd, positive clusters appeared in the left eye region. (C_1_ = 16,698.9, *p* < .001; C2 = 17,803.7, *p* < .001; C_4_ = 3062.6, *p* < .001). The maps show negative clusters (i.e., reduced P1 amplitudes) in the area of the mouth and chin for spatial frequencies between 1.3 and 2.7 cpd. (C_3_ = 14,473.1, *p* < .001; C_5_ = 2048.8, *p* = .007). For neutral faces, positive clusters appear around the right eye at a spatial frequency of 0.67 cpd. At a spatial frequency of 1.3 cpd and 2.7 cpd, positive clusters appear in the region of the inner canthus of the left eye (C_1_ = 6093.3, *p* = .010; C_2_ = 10,014.9, *p* < .001; C_4_ = 2134.4, *p* = 006). The maps show negative clusters for the right corner of the mouth for a spatial frequency of 1.3 cpd (C_3_ = 7176.6, *p* = .001). For fearful faces, significant positive clusters were observed in the region of both eyes at a spatial frequency of 0.67 to 1.3 cpd. (C_1_ = 9622.0, *p* = .003; C_2_ = 13,752.8, *p* < .001). A negative cluster can be viewed in the region of the mouth at a spatial frequency of 1.3 cpd (C_3_ = 7708.8, *p* < .001).

Again, no significant clusters were observed for the difference between fearful and neutral classification maps. For the five clusters found for the right P1, Bayesian t-tests revealed moderate evidence for the null hypothesis (C_1_: BF_01_ = 6.592, C_2_: BF_01_ = 7.465, C_3_: BF_01_ = 6.673, C_4_: BF_01_ = 7.25, and C_5_: BF_01_ = 5.867).

Average classification maps are provided in Supplementary Fig. 1. To further illustrate the relationship between local image intensity and ERPs, we extracted the image intensity for each trial and each spatial scale at the pixel with the highest absolute t-value per cluster. These intensity values were then assigned to three bins of equal contrast range, referred to as low, medium, or high-intensity trials. The resulting ERPs per intensity level illustrate how the relative visibility of the maximally effective pixel influences the ERP time course (see Supplementary Fig. 4).

#### N170

**Figure 5:**
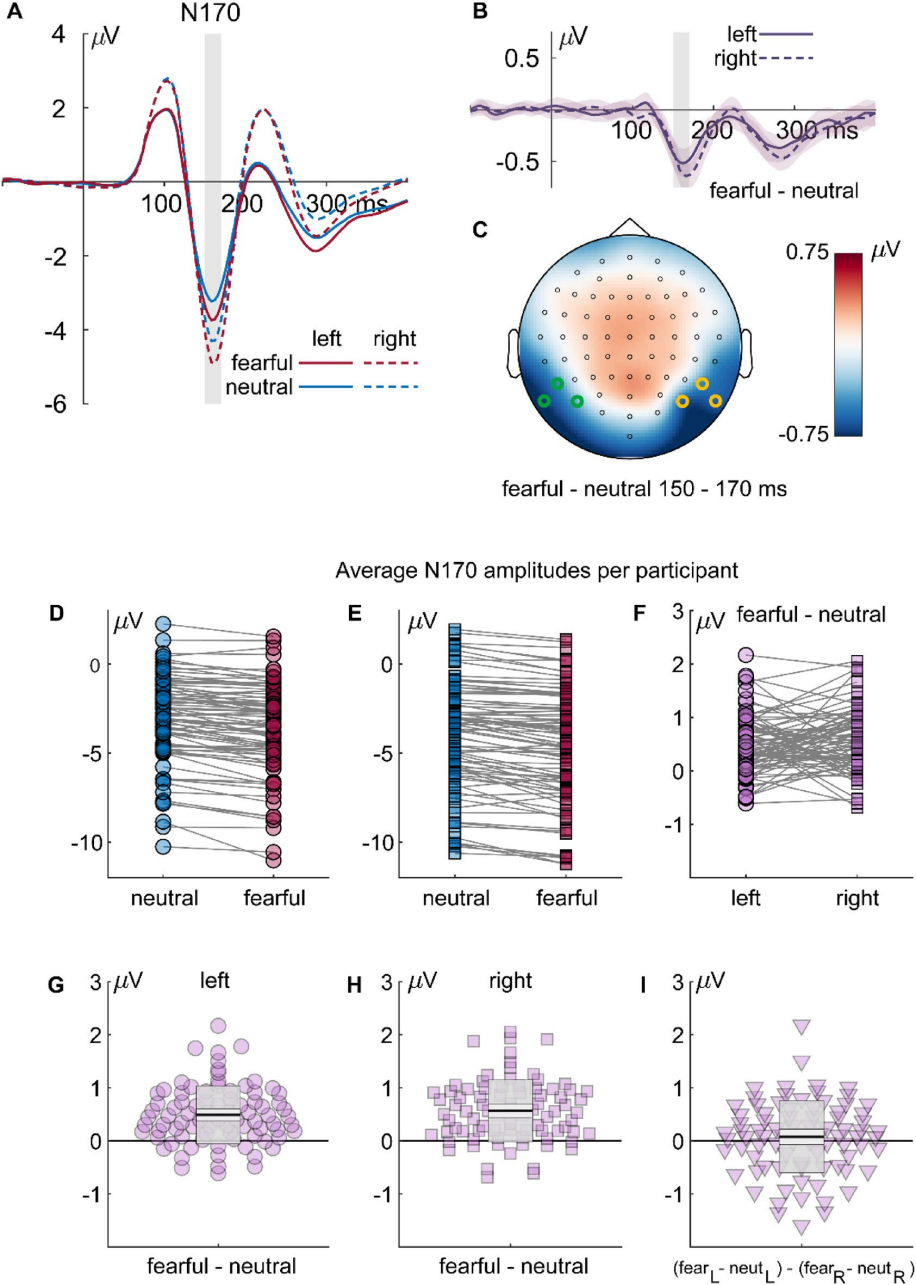
Results for the N170 component: (**A**) ERP waveforms for all fearful (red) and neutral (blue) expressions, separate for left (solid lines; P7, P9, and PO7) and right (dashed lines; P8, P10, and PO8) electrodes. (**B**) Differential ERPs (fearful–neutral; purple), separate for left (solid lines) and right (dashed lines) electrodes. The gray rectangles mark the interval of interest used for calculating average amplitudes and for the topography. Shaded areas around the differential ERPs depict the 95% bootstrap confidence intervals around the mean difference. (**C**) Topography of the differences in the highlighted interval of interest. (**D**–**F**) individual average N170 amplitudes or amplitude differences, respectively. (**G**, **H**) Individual differences between fearful and neutral faces and (**I**) individual left/right differences of fearful/neutral differences. The dots are horizontally displaced proportional to the local density to reduce cluttering. The superimposed gray boxes illustrate ± 1 standard deviation (SD; dark gray) and ± 1 standard error of measurement (SEM; light gray) around the mean (black line).

The ANOVA of mean ERPS, showed an significant main effect of emotion, F_(1,82)_ = 113.614, *p* < .001, partial *η*^2^ = .581, as well as an effect of hemisphere, F_(1,82)_ = 11.022, *p* = .001, partial *η*^2^ = .118. N170 amplitudes were larger (i.e., more negative) for fearful than neutral stimuli and larger in the right than the left hemisphere (see Fig. 5a–c). No interaction was observed. Figure 5d–i additionally illustrates individual amplitudes and amplitude differences.

**Figure 6:**
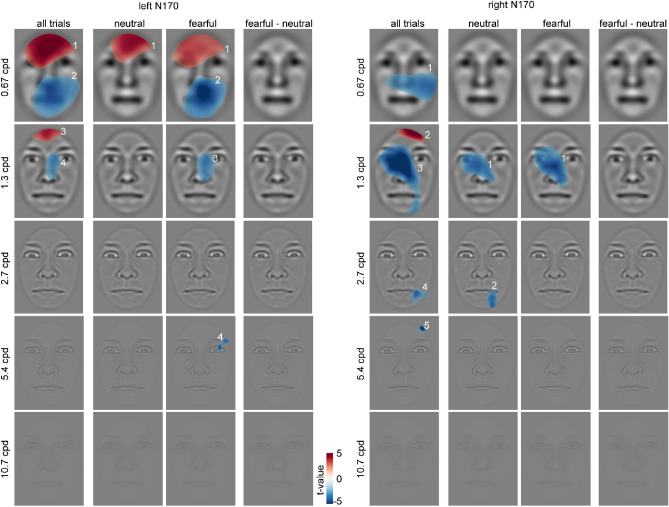
Classification images for the N170 component depicting face regions that contributed significantly to an amplitude increase (red) or decrease (blue). The clusters are enumerated to assign them to the respective statistics reported in the main text. Please note that the electrodes used for classification image calculations were chosen individually (see “Methods” section), whereas the ERP data in Fig. 5 are based on common sensors for illustration.

Classification maps for the left-hemispheric N170 of all trials show positive clusters (i.e., reduced N170 amplitudes) in the region of the forehead, the left eyebrow and the forehead, restricted to spatial frequencies between 0.67 and 1.3 cpd (C_1_ = 22,588.8, *p* < 0.001; C_3_ = 4243.3, *p* = 0.008; see Fig. 6). For all trials, negative clusters (i.e., increased N170 amplitudes) can be observed in the region of the mouth, the nose, and the left cheek at spatial frequencies between 0.67 and 1.3 cpd. (C_2_ = 23,128.5, *p* < .001; C_4_ = 4251.9, *p* = .009). For neutral faces, the maps show positive clusters in the area of the forehead and hairline at a spatial frequency of 0.67 cpd (C_1_ = 13,971.2, *p* < .001). For fearful faces, positive clusters can be observed in the region of the forehead and the left eyelid at a spatial frequency of 0.67 cpd (C_1_ = 17,506.3, *p* < .001). The maps show negative clusters in the region of the mouth, the bridge and tip of the nose and the right eyelid, restricted to spatial frequencies between 0.67 cpd and 5.4 cpd. (C_2_ = 23,595.8, *p* < .001; C_3_ = 5778.6, *p* = .001; C_4_ = 1027.2, *p* < .001). No significant clusters were observed for the difference between fearful and neutral classification maps for the left N170. For the four clusters found across all trials, we observed moderate evidence for the null hypothesis (C_1_: BF_01_ = 4.029, C_2_: BF_01_ = 7.852, C_3_: BF_01_ = 6.27, and C_4_: BF_01_ = 8.229).

For the right N170 of all trials, classification maps show positive clusters in the region of the right hairline at a spatial frequency of 1.3 cpd (C_2_ = 4108.9, *p* = .008). The maps further show negative clusters in the region of the right cheek, the left eye, the right corner of the mouth and the right hairline, at spatial frequencies between 0.67 cpd and 5.4 cpd (C_1_ = 14,785.2, *p* < .001; C_3_ = 23,455.7, *p* < .001; C_4_ = 2037.5, *p* = .006; C_5_ = 667.9, *p* = .006). For neutral faces, negative clusters can be observed in the region of the left eye, the nose and the right chin at spatial frequencies between 1.3 cpd and 2.7 cpd. (C_1_ = 10,170.3, *p* < 0.001; C_2_ = 2339.7, *p* = .003). For fearful faces, the maps show negative clusters in the region of the right eye and the nose at a spatial frequency of 1.3 cpd.

No significant clusters were observed for the difference between fearful and neutral classification maps for the right N170. For four out of the five clusters found for the right N170, we observed moderate evidence for the null hypothesis (C_1_: BF_01_ = 8.114, C_2_: BF_01_ = 4.108, C_3_: BF_01_ = 6.072, and C_5_: BF_01_ = 7.532) and anecdotal evidence for one cluster (C_4_: BF_01_ = 2.695). Average classification images and ERPs separated by intensity at the cluster maximum are provided in Supplementary Figs. 2 and 5, respectively.

#### EPN

**Figure 7:**
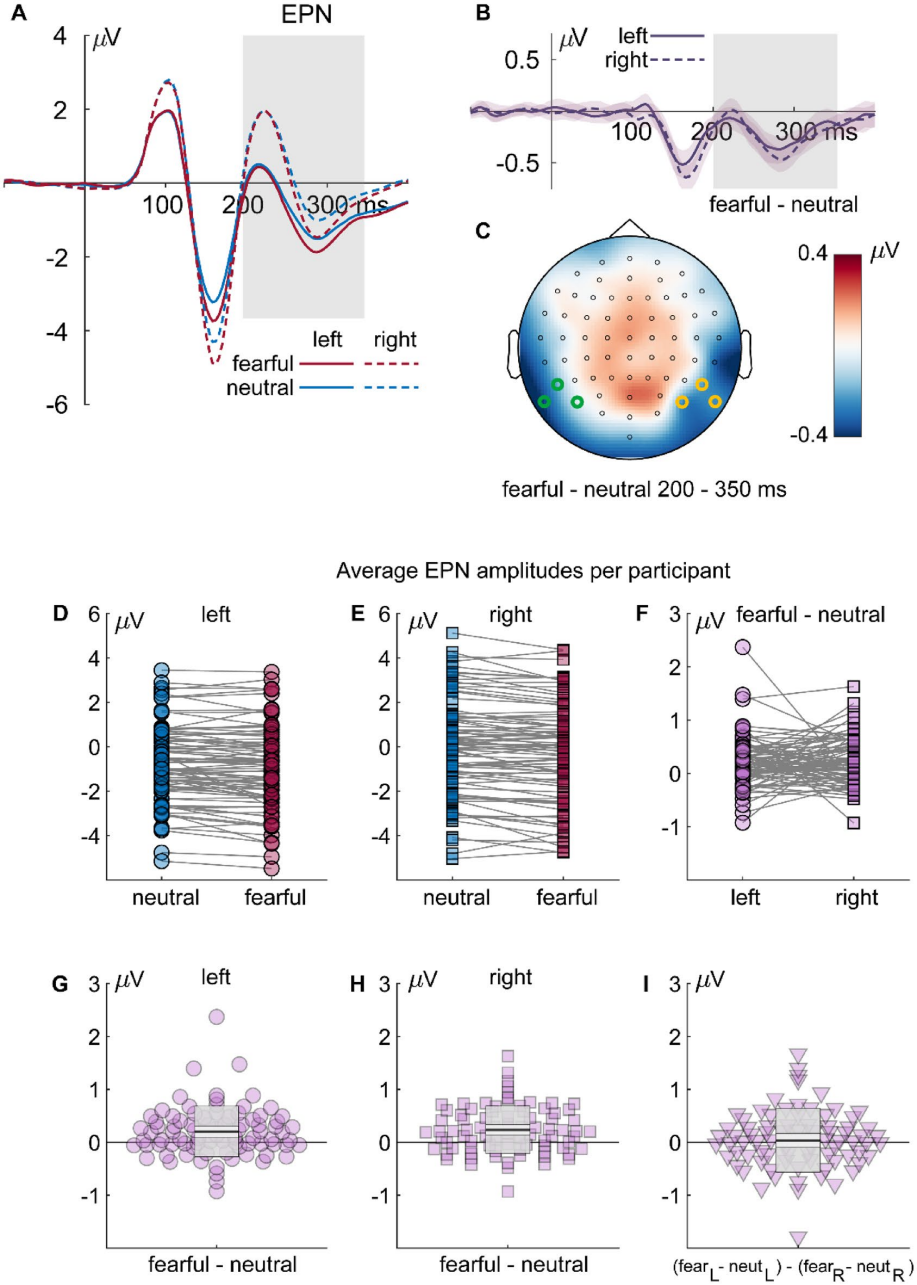
Results for the EPN component: (**A**) ERP waveforms for all fearful (red) and neutral (blue) expressions, separate for left (solid lines; P7, P9, and PO7) and right (dashed lines; P8, P10, and PO8) electrodes. (**B**) Differential ERPs (fearful–neutral; purple), separate for left (solid lines) and right (dashed lines) electrodes. The gray rectangles mark the interval of interest used for calculating average amplitudes and for the topography. Shaded areas around the differential ERPs depict the 95% bootstrap confidence intervals around the mean difference. (**C**) Topography of the differences in the highlighted interval of interest. (**D**–**F**) individual average EPN amplitudes or amplitude differences, respectively. (**G**, **H**) Individual differences between fearful and neutral faces and (**I**) individual left/right differences of fearful/neutral differences. The dots are horizontally displaced proportional to the local density to reduce cluttering. The superimposed gray boxes illustrate ± 1 standard deviation (SD; dark gray) and ± 1 standard error of measurement (SEM; light gray) around the mean (black line).

The ANOVA revealed a significant main effect of emotion , F_(1,82)_ = 32.395, *p* < .001, partial *η*^2^ = .283, and of hemisphere, F_(1,82)_ = 12.421, *p* < .001, partial *η*^2^ = .132. EPN amplitudes were larger (i.e., more negative) for fearful than neutral stimuli and larger in the left than the right hemisphere (see Fig. 7a–c). No interaction was observed. Figure 7d–i additionally illustrates individual amplitudes and amplitude differences.

**Figure 8:**
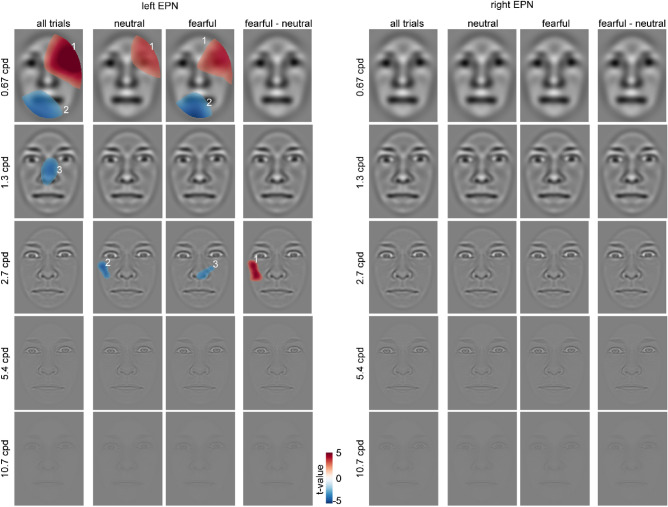
Classification images for the EPN component depicting face regions that contributed significantly to an amplitude increase (red) or decrease (blue). The clusters are enumerated to assign them to the respective statistics reported in the main text. Please note that the electrodes used for classification image calculations were chosen individually (see “Methods” section), whereas the ERP data in Fig. 7 are based on common sensors for illustration.

Classification maps for the left-hemispheric EPN of all trials show a positive cluster (i.e., reduced EPN amplitudes) in the region of the right eye and forehead at 0.67 cpd (C_1_ = 25,840.6, *p* < .001; see Fig. 8). Negative clusters (i.e., increased EPN amplitudes) appear in the left chin region and the nose at 0.67 and 1.3 cpd, respectively (C_2_ = 11,904.5, *p* = .001; C_3_ = 5514.1, *p* = .003). For neutral trials, a positive cluster was observed in the region of the right eye and forehead at 0.67 cpd (C_1_ = 12,109.3, *p* < .001) and a negative cluster at the left temple (C_2_ = 2188.7, *p* = .004) at 2.7 cpd. Fearful trials showed a similar positive cluster in the region of the right eye and forehead (C_1_ = 14,835.7, *p* < .001) and a negative cluster in the left chin region (C_1_ = 10,478.1, *p* = .002) at 0.67 cpd. Another negative cluster was found in the nose region at 2.7 cpd (C_3_ = 1868.2, *p* = .008). Finally, for the difference between fearful and neutral trials, a positive cluster (i.e., reduced EPN amplitudes for fearful compared to neutral faces) was observed at the left temple region (C_1_ = 3784.5, *p* < .001). For two of three clusters found across all trials for the left EPN, we observed moderate evidence for the null hypothesis (C_1_: BF_01_ = 7.038, and C_3_: BF_01_ = 8.158) and anecdotal evidence for one cluster (C_2_: BF_01_ = 2.11). No significant clusters were observed for the right hemispheric EPN. Average classification images and ERPs separated by intensity at the cluster maximum are provided in Supplementary Figs. 3 and 6, respectively.

### Correlations with trait anxiety

**Figure 9:**
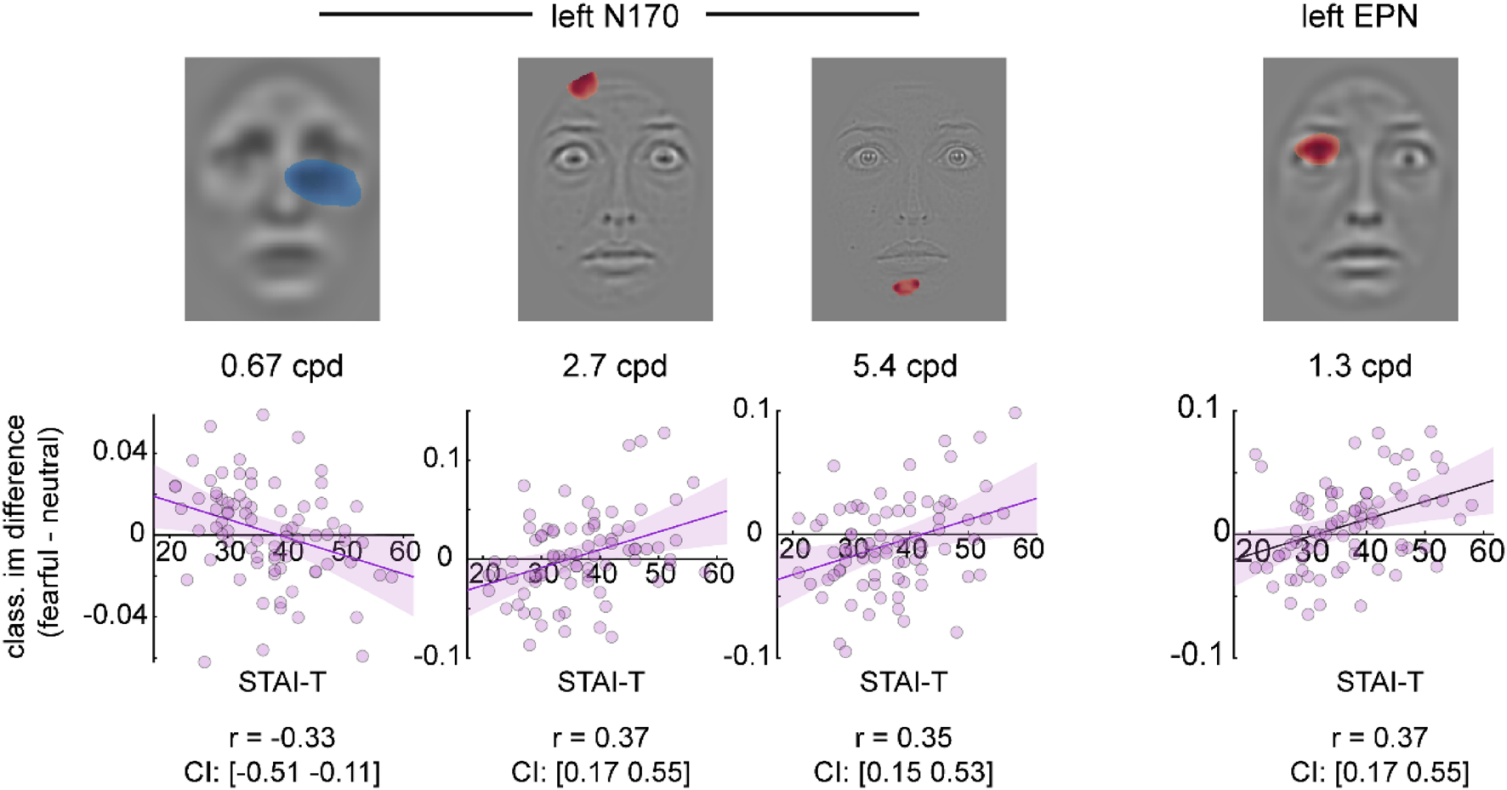
Results for cluster-based permutation analysis of correlations between trait anxiety scores and classification maps. Please note that none of the depicted clusters cross the significance threshold of cluster-α = .01. They are merely presented for completeness and future exploration. The bottom row shows scatterplots of individual trait anxiety scores plotted against the individual classification image differences (fearful–neutral) at the peak pixel of the depicted cluster. The Pearson correlation coefficient r and its confidence interval (CI) is noted below each scatterplot. Dark purple lines are linear regression lines displayed with a 95% CI.

Correlation coefficients were computed between the differential classification maps (fearful – neutral) per component and scale. Cluster-based permutation tests of these correlations did not yield any significant clusters. However, some clusters approached the critical cluster-α = .01. The coefficients ranged between r = − .451 and r = .507 at the level of individual pixels. Given our exploratory approach, we refrain from interpreting these clusters. For the sake of completeness and future studies, we report all clusters below cluster-α = .05. Please note that this involves apparently implausible face regions (e.g., the upper edge of the forehead; see Fig. 9).

For the left-hemispheric N170, we observe a negative cluster beneath the contralateral eye region at 0.67 cpd (C = 478.9, *p* = .025). The higher the trait anxiety score, the more the right eye region contributes to an increased (i.e., enhanced negativity) of the contralateral N170. For the same component, we further observe two positive clusters at 2.7 cpd located at the upper edge of the forehead (C = 116, *p* = .043) and at 5.4 cpd located at the center of the chin (C = 64.4, *p* = .014). The higher the trait anxiety score, the more these regions contribute to a decreased (i.e., reduced negativity) of the left-hemispheric N170.

For the left-hemispheric EPN, we observe a positive cluster located at the ipsilateral eye at 1.3 cpd (C = 210.38, *p* = .043). The higher the trait anxiety score, the more the left eye contributes to a reduction (i.e., reduced negativity) of the ipsilateral EPN.

## Discussion

In the present study, we investigated the role of specific face parts for the potentiation of components of the ERP to fearful compared to neutral faces. We found that different facial regions, particularly the eyes and mouth region, contributed significantly to the amplitudes of all ERP components across facial expressions. When comparing fearful and neutral faces, however, we found evidence for the absence of facial decoding effects. We also did not detect statistically significant associations between the ERP components, the classification analysis, and trait anxiety scores. These results suggest that the increased amplitudes of early ERPs to fearful compared to neutral faces are not determined by specific face regions or trait anxiety-dependent interindividual differences in face processing,

Results for the analysis of P1 amplitudes showed a specific interaction of emotion and hemisphere. In previous studies, results on P1 modulations by fearful compared to neutral faces were mixed^7^, with studies reporting differences^35,38,83^ while other studies did not observe such effects^44,58,70^. Notably, P1 differences between fearful and neutral faces are strongly driven by low-level differences between stimuli^41^, including spatial frequency^8^. The classification maps revealed that P1 amplitudes—as a whole—were enhanced by low spatial frequency information stemming from the nose and the contralateral eye region. Further, they were attenuated by low spatial frequency information from the mouth region. However, the modulation of P1 amplitudes by emotional category could not be attributed to specific face regions. Thus, this pattern adds to the mixture of fragile emotion effects on the P1 but does not single out specific facial regions responsible for these effects.

For the N170, we found that the amplitudes were more negative for fearful than neutral facial stimuli. This is consistent with a recent meta-analysis which showed that N170 is reliably modulated by fearful expressions^6^ and our own recent studies^8,43,59,70,83^. In addition, across all faces, our results replicate a specific role of the eye region for the N170. Regardless of neutral or fearful facial expressions, the eye region led to larger N170 amplitudes for all faces. This finding is in line with studies showing that N170 amplitudes are increased when only core face features are presented^59^ and, more specifically, when the eyes are fixated^14,15,55,56^. This effect has been attributed to the specific role of the eyes in social communication^84^.

However, our results suggest that no specific facial region contributes to the N170 difference between fearful and neutral faces. This finding is in contrast to studies suggesting that the eye regions influence N170 differences between fearful and neutral expressions depending on fixated faces parts^14,15^, that the white of the eye affects brain responses^85,86^, or that the eye regions support the detection of fearful faces^64^. However, our results show that across a large sample, differences in the eye or other regions are not the discriminative feature for early ERP differences during a design in which faces are task-irrelevant.

Besides the N170, also the EPN was larger for fearful compared to neutral faces in accordance with various previous studies^27,44,59,87,88^, confirming that the EPN differentiates between fearful and neutral faces in a paradigm that does neither direct attention away from the stimuli^27^, nor requires attentional focus on the emotional expression^70^. Classification maps reveal that especially the left-hemispheric EPN is sensitive to facial features and that this sensitivity is restricted to low spatial frequencies. Interestingly, the right hemispheric EPN did not reveal any dependence on specific face parts, although fearful-neutral differences were descriptively more pronounced compared to the left hemispheric EPN (see Fig. 7b). Facial regions also had no effect on this differential ERP modulation. The observation that the right hemispheric EPN is at least as expression-selective as the left hemispheric EPN, but is not driven by specific face parts, suggests that the right EPN reflects a more holistic form of face processing than the left EPN. It also suggests that holistic face processing does not come at the cost of expression selectivity.

Our findings of potentiated brain responses to threat-related faces independently from specific faces parts are in line with studies in which neutral faces were associated with threat-related or neutral information leading to P1^89,90^, N170^16,17,89,91^, or EPN^16,17^ potentiations to threat-associated faces despite identical facial features. These studies suggest that ERP modulations can be influenced by the emotional relevance of faces, independent of sensory differences between stimuli.

Our study supports the thesis that the modulation of early components, mainly of the N170 by negative facial expressions, is based on a form of holistic processing. This does not rule out a prominent role of facial features in the modulation of the N170, such as the mouth^50–54^ or eye region^53–56^. However, our study suggests that even for fearful faces, the eyes per se do not produce the emotional N170 effect, at least in designs in which faces are task-irrelevant. Specific face features might support behavioral responses and be associated with N170 amplitudes in other designs^54,66,68,69^.

Finally, there were no significant correlations between trait anxiety and the differential classification images. This implies that there are no face regions that play more important roles in high-anxious individuals compared to low-anxious individuals, at least with the statistical thresholds in the present study. Future hypothesis-driven studies might use the presented data to investigate whether some numerically high correlation coefficients are replicable (for example, between the eye-region-driven N170 and trait anxiety). Trait anxiety has been suggested to relate to different possible attentional biases^21,92–94^. ERP findings, however, remain mixed. Some studies reported a relationship between trait anxiety and increased early ERPs to fearful faces^23–25^, while other studies showed attenuated differences during mid-latency processing stages^28,29^, both effects^24^, or no relationship of the P1, N170, or EPN amplitudes and trait anxiety^26,27^. It might be that neural responses can be more reliably observed when comparing extreme groups of high versus low subclinical anxiety^95^ or in clinical samples^96^. Furthermore, fearful faces might elicit less threat-related responses, and therefore, fear-conditioning might be more potent in revealing relationships between ERP differences and trait anxiety (see^93^, but see^94^). Finally, other measures of individual differences in threat responses could reveal more systematic relationships between fear processing and anxiety, such as trait fearfulness (see Panitz et al., 2018) or other validated measures of anxiety (e.g., the STICSA, see^96^, or the GAD-7, see^97^).

We would like to note some limitations of our study and suggestions for future studies. Some points were already discussed. We investigated responses during a specific face-unrelated task; other tasks might yield other findings. It would be interesting to compare emotion detection tasks and other tasks and to better understand the role of specific features in detection performance. We used only one specific trait anxiety questionnaire and an unselected sample in an exploratory approach. Future studies might extend the investigation of interindividual differences.

## Conclusion

In this study, we investigated the contributions of facial areas to differential amplitudes of the P1, N170, and EPN to fearful vs. neutral faces. We found that different facial regions, especially the eyes and mouth regions, led to increased ERP amplitudes to all faces, while we did not detect contributions of specific face regions to ERP differences between fearful and neutral faces. There was also no modulation of findings by trait anxiety. Thus, it can be concluded that the increased amplitudes of the N170 and EPN to fearful compared to neutral faces are not due to specific facial regions and are not driven by differences in trait anxiety scores.

## Supplementary Information

Supplementary Information.

## Data Availability

All data and stimuli are available at osf.io/n72w3. All stimuli were generated from images taken from the Radboud Faces Database^73^.
