# Supplementary material for: Potentiated early neural responses to fearful faces are not driven by specific face parts
Source: Sci Rep. 2023 Mar 21;13:4613. doi: 10.1038/s41598-023-31752-z (PMC10030637; doi:10.1038/s41598-023-31752-z)
Supplement: Supplementary file 1 — Supplementary Information. [file 41598_2023_31752_MOESM1_ESM.docx]

Supplementary Materials

Please note that all face images shown here do not belong to the actual stimulus set. For purposes of copyright protection we used an artificially created face (generated using the software FaceGen 3D Print Home 2.0) as a background image for all classification maps.

**Unthresholded classification images**


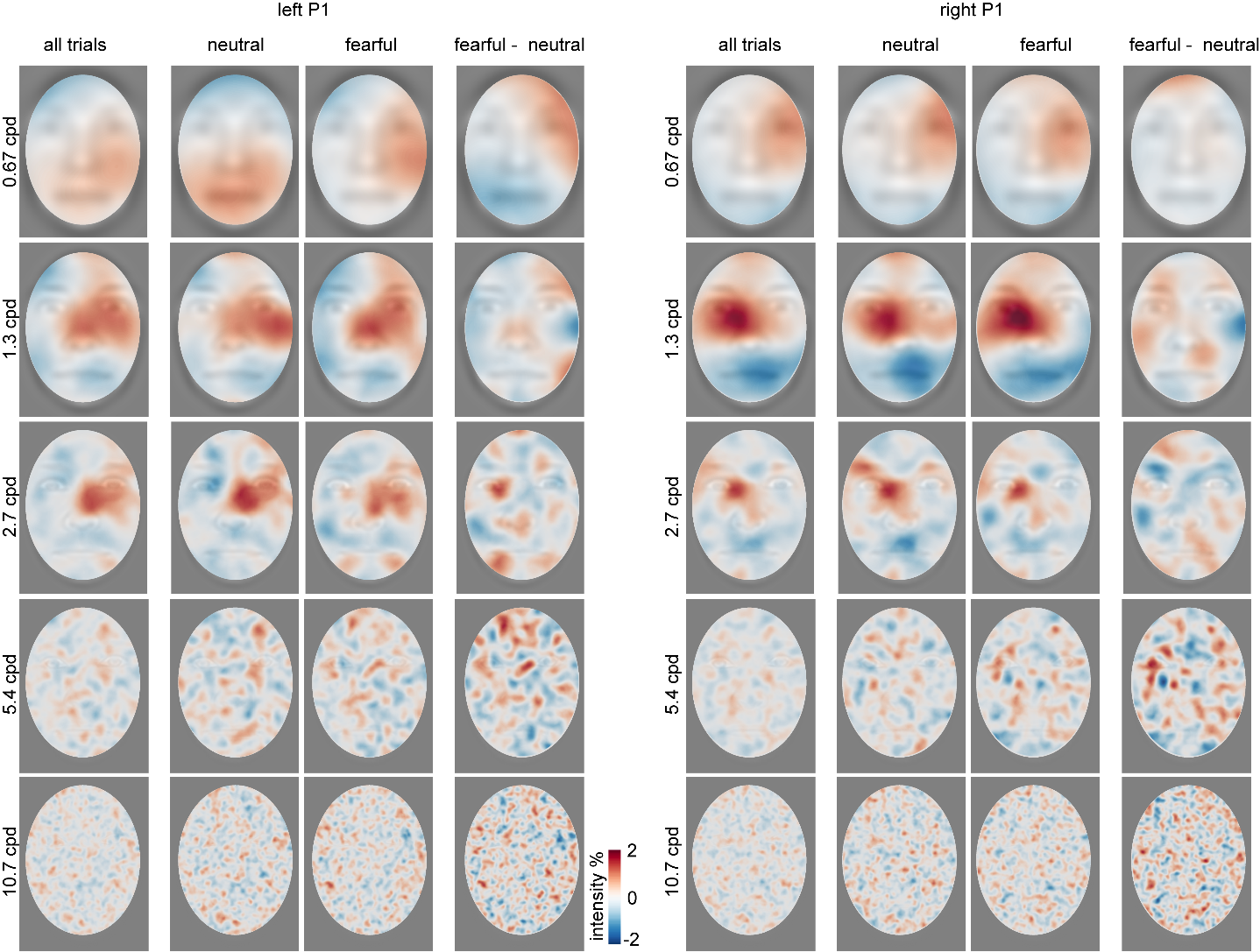


Supplementary Figure 1: Average classification images for the P1.


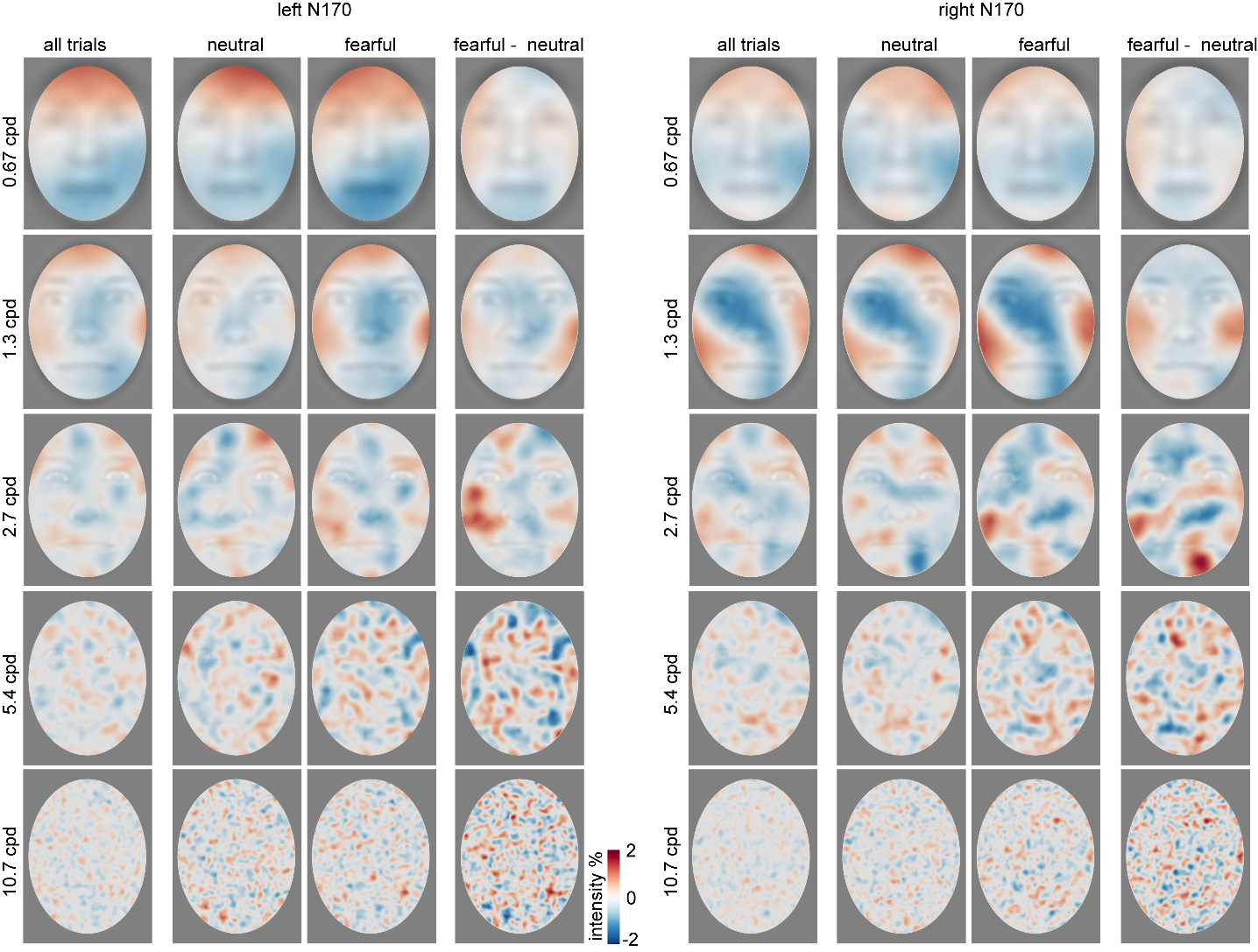


Supplementary Figure 2: Average classification images for the N170.


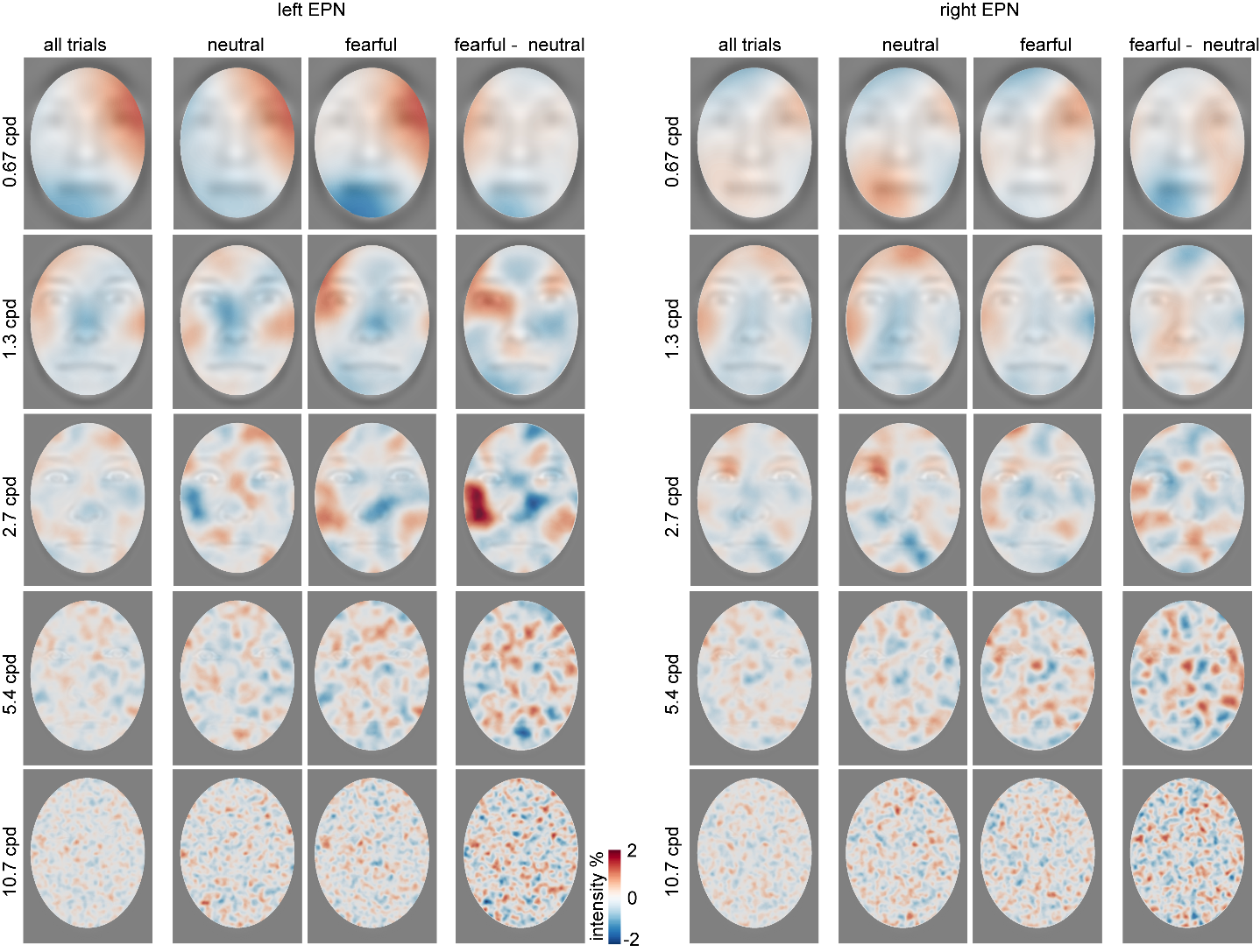


Supplementary Figure 3: Average classification images for the EPN.

**ERPs split by contrast at cluster maxima**

To illustrate the effect of image contrast in the relevant face regions on ERPs, we used the pixel intensity per trial at the location of the maxima of each significant cluster observed for classification images across all trials. For each participant we split ERPs at the individually chosen electrodes into low, medium, and high pixel intensity trials, resulting from binning intensity levels observed at the cluster maximum into three categories of equal intensity range. The resulting ERPs are reported below for the P1, N170, and EPN in Supplementary Figure 1 – 3, respectively. Please note that no inferential statistics were performed at this point, as this analysis would have been circular.


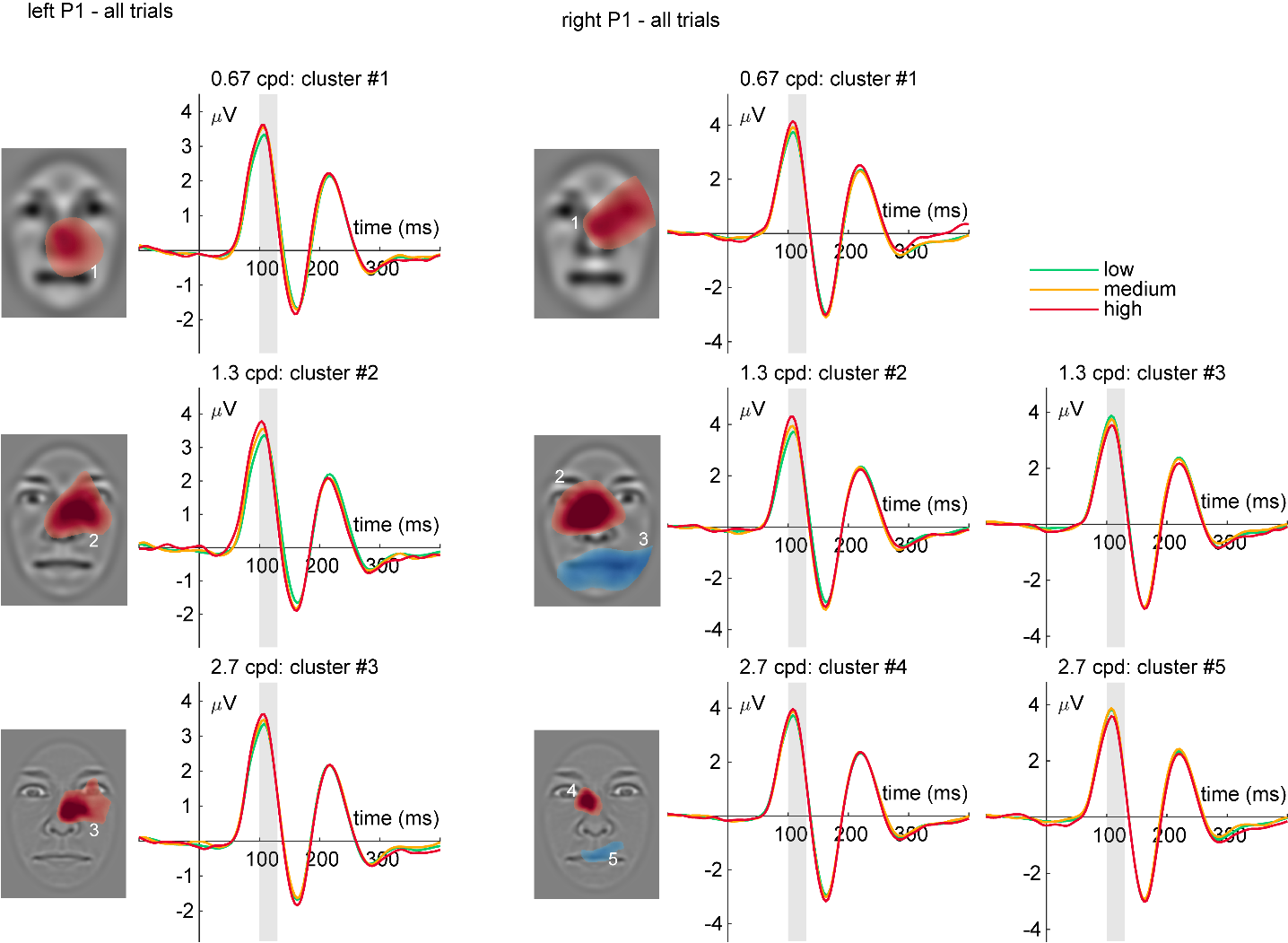


Supplementary Figure 4: ERPs at individually chosen P1 electrodes, separated by image intensity at the pixel location of each cluster’s maximum (or minimum, in case of negative clusters).


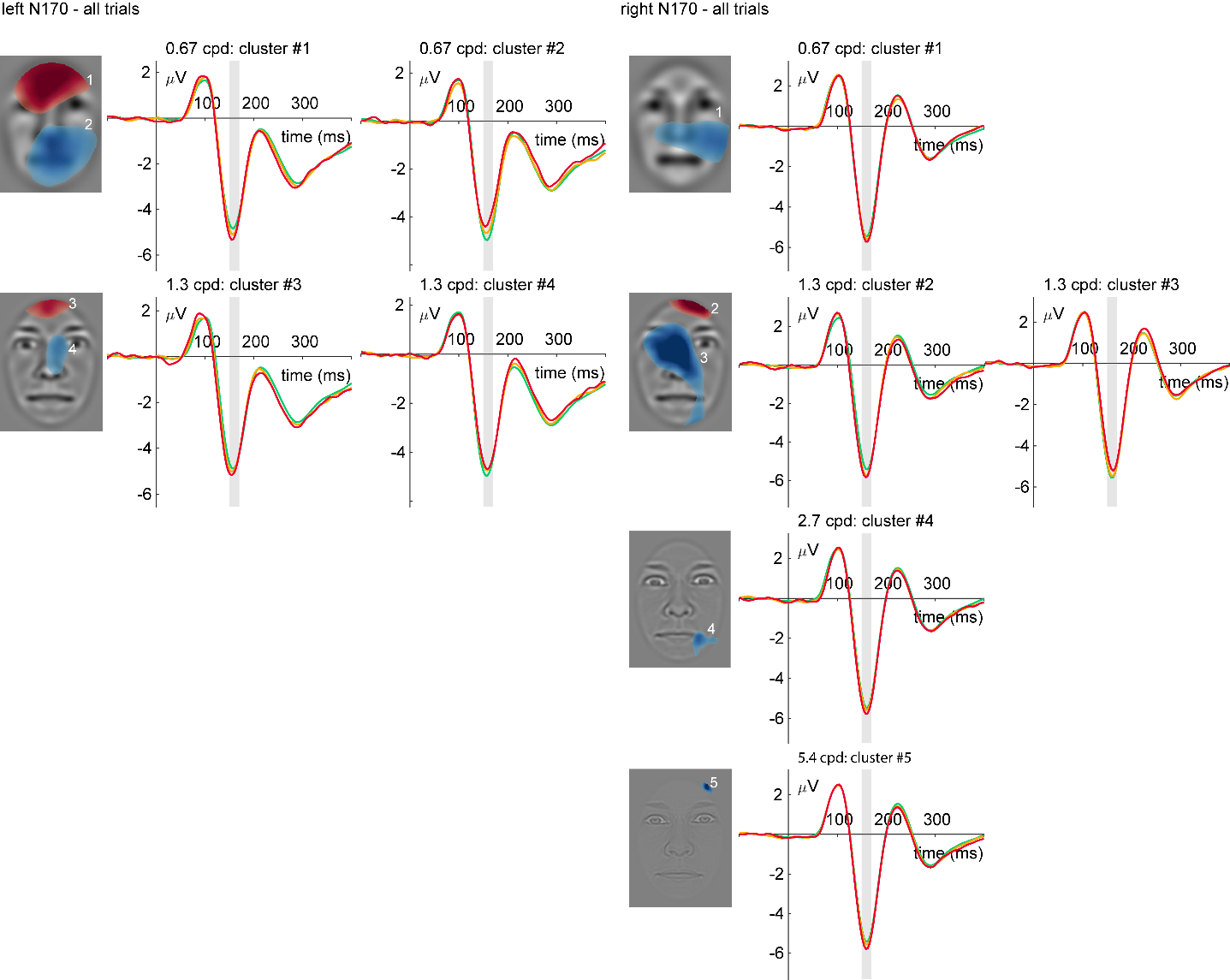


Supplementary Figure 5: ERPs at individually chosen N170 electrodes, separated by image intensity at the pixel location of each cluster’s maximum (or minimum, in case of negative clusters).


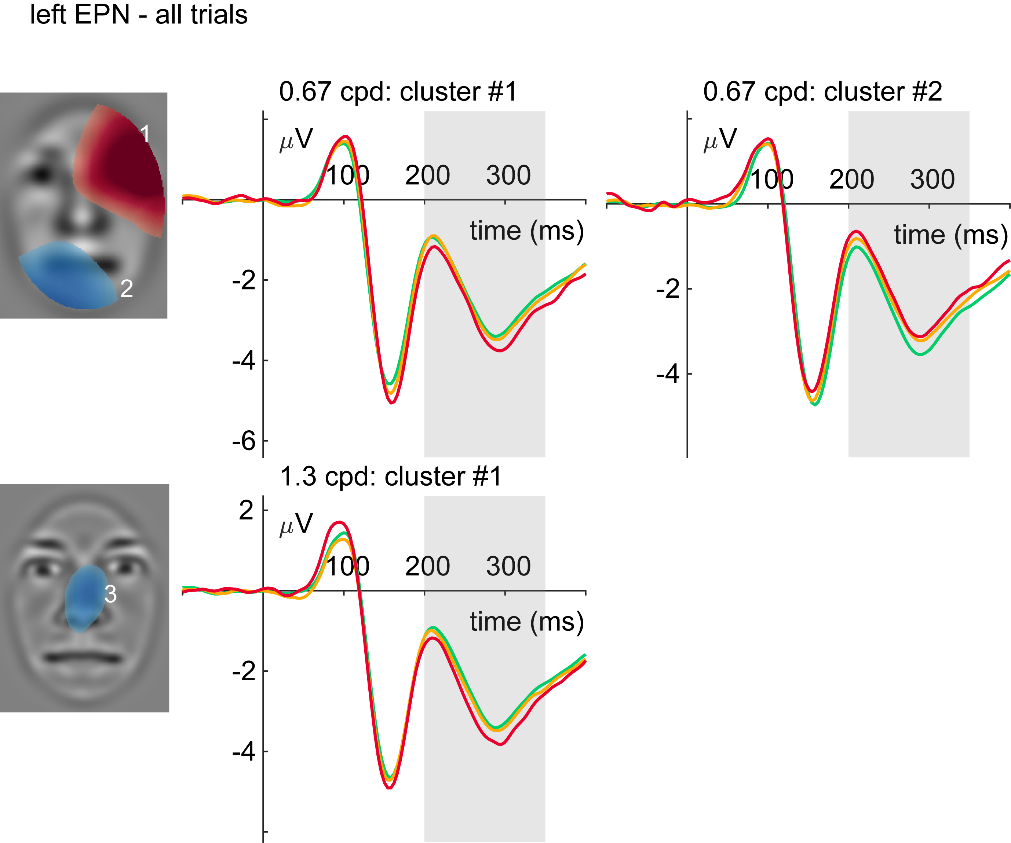


Supplementary Figure 6: ERPs at individually chosen EPN electrodes, separated by image intensity at the pixel location of each cluster’s maximum (or minimum, in case of negative clusters).
